# Laparoscopic Repair of Extraperitoneal Ureteral Inguinal Hernia With Mesh Placement

**DOI:** 10.7759/cureus.11067

**Published:** 2020-10-20

**Authors:** Ulugbek Negmadjanov, Megan Daubert, Robert D Rawlinson, Mark R Licht, Jose Yeguez

**Affiliations:** 1 Surgery, Charles E. Schmidt College of Medicine, Florida Atlantic University, Boca Raton, USA; 2 Urology, Boca Raton Regional Hospital, Boca Raton, USA

**Keywords:** ureteral inguinal hernia, hydronephrosis, laparoscopic hernia repair

## Abstract

Ureteral inguinal hernias are a well-described entity, within the spectrum of sliding hernias, with over 140 cases described since 1880. Though herniation of the ureter is relatively rare and complete ureteric obstruction is infrequent, a massive herniation may cause complete obstruction, leading to hydronephrosis. Management of these hernias is challenging and poses a significant danger of inadvertent injury and entrapment of a tortuous ureter. When faced with this type of hernia, extreme care should be taken to perform the appropriate preoperative workup and thoroughly plan the surgical approach. The present case describes a patient with a known ureteral inguinal hernia, who underwent a laparoscopic repair of the hernia with mesh placement.

## Introduction

Ureteral inguinal hernias more commonly occur in men in the fifth and sixth decades of their life [1]. These hernias occur predominantly on the right side because, on the left side, the fascia of Toldt sits at the root of the sigmoid colon mesentery, which fixes the ureter in the retroperitoneum [2]. Ureteral hernias are predominantly indirect, and incarceration is relatively infrequent due to the hernia's large size [3]. 

Though some of these patients may present with enlarging scrotum [4], unexplained urinary tract infection, hydroureter, or hydronephrosis [5], they often go undiagnosed until a surgical repair is performed. This presentation certainly increases the intraoperative risk of injury to the ureter with an increased risk of mesh infection if one is placed. Thus, it is highly essential to maintain a high degree of suspicion when patients present with inguinal hernia and unexplained obstructive uropathy or frequent urinary tract infections. If such a patient is identified preoperatively, a CT scan with urogram can be utilized to delineate the anatomy better and plan the management approach. The surgical approach in treating these hernias usually consists of ureteral protection with cystoscopy and stent placement, followed by open repair and reduction. The laparoscopic approach has been previously described in the management of urinary bladder hernias [6], but to our knowledge, there are only several case reports describing a minimally invasive approach for repair of ureteral hernias [7-9]. Our case report shows that, in situations where a robotic system is not available, a laparoscopic approach combined with distal stenting of the ureter may be a better option to decrease the risk of injury to the ureter. Implementation of this approach allowed us to minimize the risk of injury to the ureter, as well as the risks of the surgical site and mesh infection. 

## Case presentation

**Figure 1:**
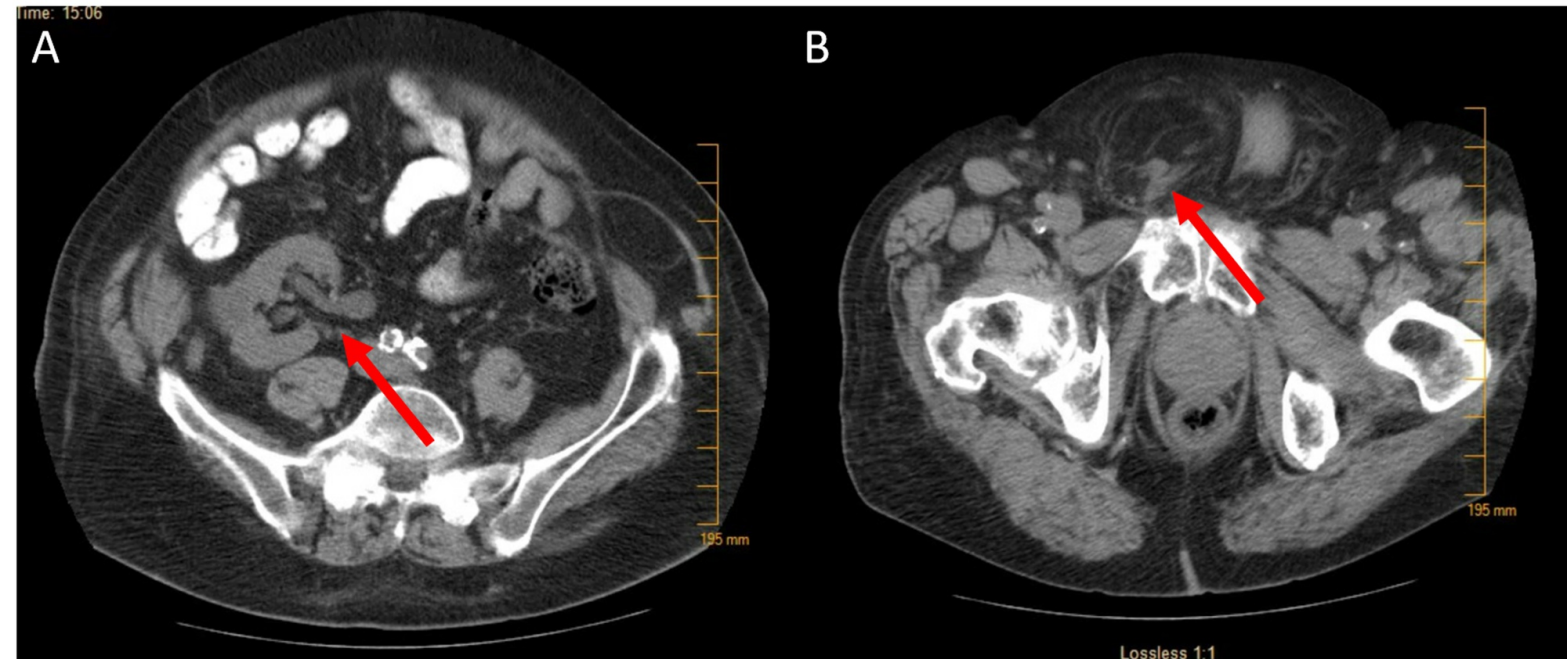
Preoperative CT scan of the abdomen and pelvis A: Right nephroptosis and hydronephrosis (red arrow). B: Right ureter with preperitoneal fat in the scrotum (red arrow).

An 87-year-old man was referred to the office by a urologist with a chief complaint of right groin pain and enlarging scrotal bulge that had been present for two months. He initially sought urologic consultation with the same complaint, as he already had an established relationship with his urologist. His medical history was notable for hypertension, hypothyroidism, and prostate cancer, for which he underwent radiation treatment three years prior. His surgical history was notable for an open left inguinal hernia repair with mesh and laparoscopic cholecystectomy. On physical exam, the patient had chronically incarcerated right inguinal hernia that was not reducible. The patient's laboratory workup was unremarkable with a normal renal function and a normal urinalysis. An outpatient CT scan one month before evaluation had shown a right nephroptosis, hydronephrosis, and hydroureter (Figure 1A) with part of the right ureter inside the right inguinal canal (Figure 1B).

**Figure 2:**
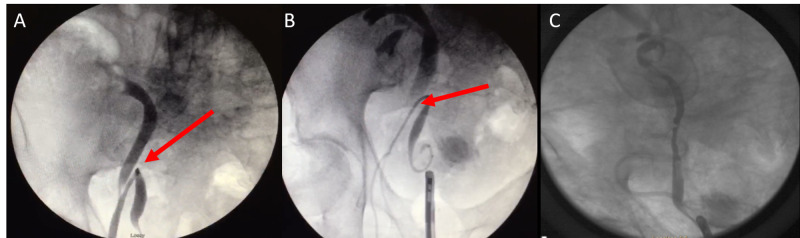
Preoperative and intraoperative retrograde pyelogram A: Angulated and tortuous ureter with the distal end within the scrotum (red arrow). B: Ureteral stent deployed in the distal ureter (red arrow). C: Completely reduced ureter.

After discussing management with the patient, the decision was made to proceed with laparoscopic repair of the hernia with preoperative cystoscopy, retrograde pyelogram, and ureteral stent placement. The patient was placed in a lithotomy position, and cystoscopy with a retrograde pyelogram was performed. The ureter looped down into the scrotum (Figure 2A) and then turned back up into the retroperitoneum into its normal anatomic position. Wire passage was attempted into the renal pelvis but was unsuccessful due to significant angulation and tortuosity of the ureter. A 6 mm x 30 mm Polaris stent (Boston Scientific, Marlborough, MA) was deployed in the distal ureter (Figure 2B). We then proceeded with the laparoscopic portion of the case by placing a 12 mm port in a supraumbilical position and two 5 mm flank ports. A peritoneal flap was developed, and a substantial amount of preperitoneal fat was observed protruding through the defect. Using gentle manual pressure and endoscopic traction, we were able to reduce the hernia sac contents. We could not directly visualize the reduced ureter, but we could identify successful reduction by fluoroscopic visualization of the reduced ureteral stent into the peritoneal cavity. Following dissection and identification of cord structures, 16 cm x 10.8 cm 3D Max Mesh (C. R. Bard Inc., New Providence, NJ) was placed over the defect. Mesh was fixed to the Cooper’s ligament medially using a Protack 5 mm device (Medtronic, Dublin, Ireland), and the peritoneal flap was closed over the mesh. After completing the laparoscopic portion of the procedure, the retrograde pyelogram demonstrated a complete reduction of the ureter into the peritoneal cavity (Figure 2C). The ureteral stent was repositioned over the guidewire.

The patient's postoperative course was uncomplicated, and he was discharged home on postoperative day two. He was recalled for his second cystourethroscopy, where his ureteral stent was removed after six weeks. A review of the patient's medical records revealed that he underwent a CT scan of the abdomen and pelvis for unrelated reasons 15 months after herniorrhaphy, which demonstrated no recurrence of the hernia with improved hydronephrosis and nephroptosis. 

## Discussion

Ureteral inguinal hernias occur in two types: paraperitoneal and extraperitoneal [10]. Paraperitoneal hernias are an acquired type of hernia, in which a herniated peritoneal sac applies traction to the ureter and brings it down [2]. In contrast, extraperitoneal hernias are thought to occur due to an embryologic defect, in which the ureteral bud does not separate from the Wolffian ducts. This results in a fusion between the ureter and genitofemoral ligaments [11]. These hernias have been described to contain large amounts of retroperitoneal fat and are associated with nephroptosis [10]. 

Most commonly, ureteral inguinal hernias occur on the right side due to a lack of reinforcement of the fascia of Toldt on the right side by a sigmoid mesentery. Certain conditions, such as kidney transplants and obesity, increase the risk of developing these hernias. Patients with ureteral herniation are often asymptomatic, and strangulation is rare given the wide fascial defect in these types of hernias. However, regardless of the etiology, surgical repair is recommended in all cases to decrease the risk of obstructive uropathy. 

While minimally invasive techniques have been previously described in the management of ureteral inguinal hernias, we demonstrate the feasibility of a laparoscopic approach combined with an endourologic approach to identify and protect the ureter intraoperatively. Laparoscopy offers advantages when compared to an open approach in this case. Apart from allowing less technically challenging combined on table retrograde pyelogram and fluoroscopy, it assists in a more rapid recovery, lower analgesic requirements, and lower recurrence rate. There are currently several described cases of robotic laparoscopic repair of ureteral inguinal hernias in the published literature, but no cases describing a laparoscopic approach. While the robotic approach combined with intraureteral use of indocyanine green (ICG) for identifying the ureter can be beneficial, in cases with chronically incarcerated hernia containing angulated and tortuous ureter, our approach might provide an alternative method of safe, minimally invasive repair when the robotic system is not available.

## Conclusions

Herniation of the ureter is a rare clinical finding with a variety of potentially significant complications. A detailed history and physical examination are keys to diagnosis. Radiological and endoscopic assessments are critical components of the preoperative and intraoperative management. Although most authors to date have employed an open approach to surgical repair of ureteral inguinal hernias and some described a robotic approach, we have confirmed that these hernias can be managed safely laparoscopically with excellent postoperative outcomes.
